# GlyCompute: towards the automated analysis of protein N-linked glycosylation kinetics via an open-source computational framework

**DOI:** 10.1007/s00216-024-05522-3

**Published:** 2024-09-26

**Authors:** Konstantinos Flevaris, Pavlos Kotidis, Cleo Kontoravdi

**Affiliations:** 1 Department of Chemical Engineering, Imperial, London, SW7 2AZ UK; 2 Present Address: Biopharm Process Research, GSK, Stevenage, UK

**Keywords:** Glycosylation, Graph theory, Kinetic modeling, Parameter estimation, Bayesian inference

## Abstract

Understanding the complex biosynthetic pathways of glycosylation is crucial for the expanding field of glycosciences. Computer-aided glycosylation analysis has greatly benefited in recent years from the development of tools found in web-based portals and open-source libraries. However, the in silico analysis of cellular glycosylation kinetics is underrepresented in current glycoscience-related tools and databases. This could be partly attributed to the limited accessibility of kinetic models developed using proprietary software and the difficulty in reliably parameterising such models. This work aims to address these challenges by proposing GlyCompute, an open-source framework demonstrating a novel, streamlined approach for the assembly, simulation, and parameterisation of kinetic models of protein N-linked glycosylation. Specifically, given one or more sets of experimentally observed N-glycan structures and their relative abundances, minimum representations of a glycosylation reaction network are generated. The topology of the resulting networks is then used to automatically assemble the material balances and kinetic mechanisms underpinning the mathematical model. To match the experimentally observed relative abundances, a sequential parameter estimation strategy using Bayesian inference is proposed, with stages determined automatically based on the underlying network topology. The proposed framework was tested on a case study involving the simultaneous fitting of the kinetic model to two protein N-linked glycoprofiles produced by the same CHO cell culture, showing good agreement with experimental observations. We envision that GlyCompute could help glycoscientists gain quantitative insights into the effect of enzyme kinetics and their perturbations on experimentally observed glycoprofiles in biomanufacturing and clinical settings.

**Graphical Abstract:**

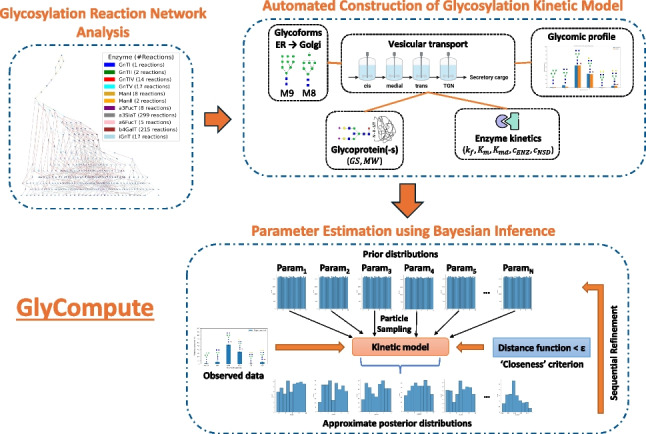

**Supplementary Information:**

The online version contains supplementary material available at 10.1007/s00216-024-05522-3.

## Introduction

In the expanding field of glycosciences, understanding the complex biosynthetic pathways of glycosylation has become pivotal for different aspects of biological research. The glycome, which constitutes the collection of oligosaccharides present in an organism, tissue, cell, or protein [1], is a critical determinant of overall molecular function and has a significant role in biomarker research for multiple disease types [2–4], understanding cellular communication [5], and efficacy and safety of glycoprotein-based biotherapeutics [6]. The study of the glycome has greatly benefited from the recent surge in data collection and algorithmic advancements, efforts that are in large part coordinated by a global partnership called GlySpace Alliance [7] in the form of bioinformatics tools hosted in centralised web portals [8–10]. These efforts complement open-source frameworks for the analysis of glycan data and their biosynthetic networks, including GNAT [11, 12], glypy [13], Glycowork [14], GlyCompare [15, 16], and GlycoMME [17]. Both web-based and open-source frameworks integrate glycobioinformatics tools that enable a diverse set of tasks, including but not limited to conversion between different glycan nomenclature notations [10, 13, 14], drawing of glycan structures [18–21] according to the Symbol Nomenclature for Glycans (SNFG) guidelines [22, 23], as well as qualitative and quantitative analyses of glycan biosynthetic networks [11, 12, 15–17, 24]. Despite these developments, the quantitative analysis of cellular glycosylation kinetics is underrepresented in current tools and databases. This could be attributed to the domain knowledge required to develop kinetic models of protein glycosylation, the limited accessibility to existing kinetic models, as they were predominantly developed and simulated using proprietary software, and the difficulty in reliably parameterising such models. Quantitative methodologies could provide insight into the cellular glycosylation machinery by quantifying the effect of enzyme kinetics on observed glycoprofiles, thus allowing glycobiologists with minimal or no modelling experience to generate experimentally testable hypotheses that could explain cellular phenotypes. To enable such analyses, the following are required: (i) a description of the glycosylation reaction network (GRN) for the glycan type of interest (e.g. N-glycans), (ii) a mechanistic model of glycosylation describing the kinetic mechanisms for the biosynthetic enzymes (i.e. glycosidases, glycosyltransferases) involved in the GRN, (iii) a parameter estimation strategy to propose plausible values for key kinetic parameters accounting for uncertainty.

When it comes to establishing GRNs, a pivotal contribution was the introduction of the automated in silico construction of GRNs by Krambeck and Betenbaugh [25]. This seminal work innovatively used sets of reaction rules that specified the necessary conditions under which each glycan-processing enzyme could catalyse a particular reaction by acting on a specific substrate. Thus, based on the number of reaction rules that were selected by the user and a starting substrate, the products of all enzymatic reactions could be inferred. This concept was powerful and scalable since the size of the glycosylation network could be traced back to the number of enzymes and their specificities. This automated GRN construction approach has been adopted by subsequent network generation frameworks, including Glycan Pathway Predictor (GPP) [26]. GPP is a web-based tool integrated within the GlySpace Alliance that allows the user to assign customisable reaction rules to generate GRNs involving both N- and O-glycans. Nevertheless, given the structural complexity of glycans and the promiscuity of glycan-processing enzymes, numerous reaction pathways can connect a single initial glycan to a specific product. Therefore, the resulting network could become cumbersome comprising a large number of biosynthetic pathways, many of which may not hold biological relevance. This was recognised independently by different research efforts, including Liu and Neelamegham [11] and Krambeck et al. [27], with the former pioneering the application of graph theoretic approaches in analysing and pruning GRNs using GNAT and the latter implementing a pruning approach to determine the dominant biosynthetic pathways based on their corresponding reaction rates using GLYMMER™ (ReacTech Inc., VA, USA).

An alternative approach to constructing GRNs without relying on explicitly formulated reaction rules was recently introduced by Thomes et al. [28, 29] in Glycowork. In particular, the comprehensive methodology considered the construction of a GRN involving milk oligosaccharides, where an adjacency matrix was initially formed, describing glycans connected through a single biosynthetic step, based on a pre-specified list of breast milk glycans. Through in silico modifications at the non-reducing ends of the observed glycan structures, unobserved intermediate structures were inferred and connections between them and the observed glycan structures were formed via an iterative approach based on shortest paths. To tackle emerging redundancies from the introduction of the inferred structures, Glycowork implemented a novel pruning strategy, termed “evolutionary pruning”, which removed less probable paths from the network by assessing the evolutionary conservation of certain glycans across mammalian species. This involved the extraction of diamond-shaped motifs with virtual nodes, assessing their presence in other milk glycome networks and their experimental validation. Paths exclusively involving unverified virtual nodes were eliminated based on the ratio of experimental observations to the total of experimental and inferred paths. The network was pruned to favour paths with significantly higher likelihoods, while maintaining overall connectivity, thus returning a biologically relevant and efficiently structured GRN.

Regardless of the pruning approach, a key challenge remains the inability to experimentally validate the actual GRN size and the presence of certain intermediate structures in vivo, since many theoretically possible reactions may be hindered by low glycoenzyme levels, limited metabolic resources, and/or steric hindrance due to the structural conformation of the protein cargo.

The development of kinetic models of protein N-linked glycosylation for a given GRN has seen several developments in the last few decades, predominantly aiming to predict the glycosylation profiles of protein-based therapeutics produced by mammalian cell cultures. These modelling efforts were recently reviewed here by Shek et al. [30]. To achieve satisfactory agreement between model predictions and experimental observations, a key challenge is the accurate estimation of model kinetic parameters. This task is non-trivial considering the large, divergent reaction networks brought about by the promiscuity of glycosyltransferases and the dependence of the model’s predictive performance on the Golgi concentration of glycosidases, glycosyltransferases, and nucleotide sugar donors (NSDs). These considerations result in a high-dimensional parameter estimation problem. In this setting, the simultaneous estimation of kinetic parameters becomes impractical and may also encounter identifiability issues [31]. To address this challenge, elaborate parameter estimation strategies have been developed [32–34], which implement a sequential estimation strategy leveraging domain knowledge about the maturation stages taking place in N-glycan biosynthesis. These strategies have been successful in deriving biologically relevant bounds for parameters, particularly enzyme concentrations [35], that lead to predictions satisfactorily matching experimental observations, thus demonstrating effective approaches to parameterising glycosylation kinetic models. However, they are not yet conducive to automated analyses by non-experts.

Regarding the optimisation methods employed for parameter estimation in existing works, approaches are primarily based on maximum likelihood estimation (MLE) using gradient-based optimisation methods. Despite the typically fast local convergence exhibited by such parameter estimation procedures, they may return locally optimal solutions, especially in highly non-linear settings such as glycosylation kinetics, thus undermining the quality of the parameter estimates. Moreover, the pairwise ellipsoidal confidence regions around parameter estimates are based on the estimation of the Fisher Information Matrix (FIM), whose accuracy depends on the linearisation of the negative log-likelihood function around the (locally) optimal model parameters, which, in turn, may not be reliable in highly non-linear settings [36]. Consequently, there is a need for a parameter estimation approach that incorporates prior knowledge and does not rely on distributional assumptions about the shape of the confidence regions, thereby robustly accounting for uncertainty.

Taking all the above into consideration, this work aims to expand upon previous modelling studies by proposing GlyCompute: a modular, open-source methodology for simulating and parameterising kinetic models of protein N-linked glycosylation keeping user decisions to a minimum. To achieve this, GlyCompute is a framework for automated kinetic model assembly based on available glycosylation pathway knowledge and formalises a Bayesian inference-based sequential parameter estimation approach leveraging domain knowledge and topological features of GRNs. We assess the effect of GRN generation and pruning approach on the agreement between model predictions and experimental observations. To the best of our knowledge, this is the first time such a consideration has been made in a kinetic modelling study of protein N-linked glycosylation. GlyCompute is showcased in a case study that considers the joint fitting of the kinetic model to the protein N-linked glycosylation profiles of intracellular host cell proteins (HCP) and a recombinant immunoglobulin G (IgG) product of CHO cell culture. The inclusion of protein-specific N-linked glycosylation pathways enables the discernment of cellular glycosylation effects on recombinant protein glycoprofiles, thus providing a comprehensive overview of N-glycan biosynthesis in CHO cells. This is a considerable advance in the development of glycosylation models that simultaneously predict the glycoprofiles of multiple protein entities from the same cell culture. We believe that methodologies demonstrating streamlined model assembly, simulation, and parameterisation of glycosylation kinetic models serve as prerequisites towards automating model-based quantitative analysis of glycomic data.

## Materials and methods

**Fig. 1:**
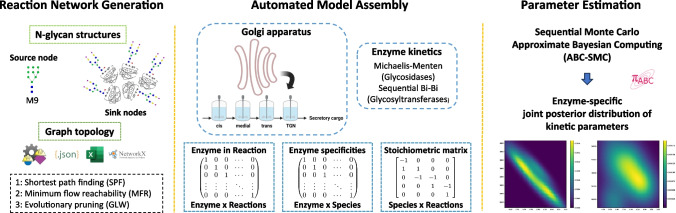
Schematic of the proposed methodological framework for building, simulating, and parameterising kinetic models of protein N-linked glycosylation

GlyCompute comprises three interconnected modules, namely the reaction network generation module, the automated model assembly module, and the parameter estimation module. A schematic of the workflow is presented in Fig. 1. All analyses were performed using Python 3.10. Unless otherwise stated, data analysis and scientific computing were carried out using *NumPy* (version 1.26.4) [37], *SciPy* (version 1.12.0) [37], and *pandas* (version 2.2.1) [38]. Glycan-related tasks, including nomenclature conversion, were carried out using *Glycowork* (version 1.2.0) [14].

### Reaction network generation

This module requires a GRN represented as a directed acyclic graph (DAG), where the nodes represent N-glycan species and the edges represent biosynthetic reactions catalysed by glycosidases or glycosyltransferases. The GRN can be generated either by pruning a larger template GRN or directly using the functionalities provided by Glycowork. In this work, CHOGlycoNET was used as a template GRN [39]. CHOGlycoNET is a comprehensive GRN tailored to CHO cells that was constructed using 200 N-glycan datasets for both natively and recombinantly produced proteins found in different CHO cell lines. CHOGlycoNET was generated by combining 200 subgraphs, one for each dataset, using the network analysis functionalities of GLYMMER™ [27]. In this work, CHOGlycoNET was pruned to generate subgraphs that can capture the connections between a source node and multiple sink nodes. M9 was selected as the source node since it is the initial glycan species that leaves the ER (alongside M8) and enters the Golgi apparatus according to the canonical secretory pathway for N-glycans [40, 41]. The experimentally observed glycan species were selected as sink nodes. The full list of N-glycan structures considered in this study can be found in the Supplementary Information.

Two approaches were considered for pruning the template GRN in this study. The first approach, termed hereafter as shortest path finding (SPF), searched for all shortest paths between the source node and each sink node using Dijkstra’s algorithm [42] and combined them to generate the subgraph mGRN_SPF_. The second approach, termed hereafter as minimum flow reachability (MFR), returned the subgraph mGRN_MFR_ including the source node and all sink nodes by ensuring that enough flow reaches all sink nodes. To achieve this, subgraph generation was formulated as a maximum single-commodity flow problem between two artificially constructed nodes, the “SuperSource” and “SuperSink” nodes. SuperSource was a node that was directly connected to the actual source node and SuperSink was a node that accepted all outflows from the actual sink nodes. The outflows from the actual sink nodes were constrained to receive a minimum required flow capacity, which would be interpreted as ensuring connectivity between each actual sink and the SuperSink nodes. The goal was to compute the maximum flow from the SuperSource to the SuperSink while ensuring that all actual sink nodes receive the minimum required flow. This would ensure that the returned subgraph would maintain connectivity between the source node and all sink nodes. All graph analysis methods were implemented using *NetworkX* (version 3.3) [43]. Finally, GRN generation was also carried out by directly implementing the network construction capabilities of Glycowork, which included evolutionary pruning, termed henceforth the Glycowork-based approach (GLW). As a first step, the GRN mediated by glycosyltransferases was generated using the experimentally observed sink nodes, and then, the GRN segment mediated by glycosidases was appended to construct the final subgraph, mGRN_GLW_.

### Automated model assembly

Once the GRN was available, an automated process to extract the protein N-linked glycosylation pathway information was carried out. This information included the stoichiometric matrix of the reaction network and enzyme specificities, which can then be used to construct the mass balances underpinning the mechanistic model of protein N-linked glycosylation, as well as describe the enzyme kinetics in the Golgi apparatus. The mathematical description of the Golgi apparatus in this work was based on the work of Krambeck and Betenbaugh [25]. The Golgi apparatus was modelled according to the vesicular transport hypothesis as a series of four well-mixed bioreactors, where each bioreactor denotes a distinct Golgi compartment, namely, the *cis*, *medial*, *trans* Golgi, and *TGN* compartments. Glycosidases were assumed to follow Michaelis–Menten kinetics and all glycosyltransferases were assumed to follow sequential-order Bi-Bi kinetics, which include the effect of NSDs on the reaction rates. The uniform assignment of sequential-order Bi-Bi kinetics to all glycosyltransferases constitutes a simplification of the kinetic mechanisms of a6FucT, GnTIII, and a3SiaT, which have been previously modelled using random-order Bi-Bi kinetics [32]. The use of simplified kinetics in this work was employed as an attempt to reduce model complexity and facilitate subsequent parameter estimation activities. It is noted, however, that assigning random-order or other kinetic mechanisms could be done with few code modifications in the underlying framework. The sequential-order Bi-Bi kinetic expressions were further modified compared to [32] by considering the competitive inhibition emerging between the N-glycans on the different protein entities (here IgG and HCP) for the same glycosyltransferases. This consideration enforces a direct dependency between recombinant protein and cellular glycosylation, thus enabling the inference of plausible kinetic parameters that could explain the observed glycoprofiles given this interplay. If model fitting using the glycoprofile of a single protein entity is of interest, then these additional inhibitory terms can be removed. The mathematical formulation is as follows:

#### Sets and indices

*I*: Set of oligosaccharide substrates as determined by the stoichiometric matrix $S$
*J*: Set of reactions as determined by the stoichiometric matrix $S$
*E*_*MM*_: Set of enzymes following Michaelis–Menten kinetics.
*E*_*BiBi*_: Set of enzymes following sequential Bi-Bi kinetics.
*K*_*e*_: Set of NSDs specific to enzyme $e\in {E}_{BiBi}$  
*P*_*ent*_: Set of protein entities.

#### Kinetic expressions

For $e\in {E}_{MM}$, the reaction rate $r_{MM,\;i,\;j,\;e,\;p}$ is given by:

1 $$r_{MM,\;i,\;j,\;e,\;p}=\frac{k_{f,\;j,\;e}{\left[ENZ\right]}_{j,\;e}{\left[OS\right]}_{i,\;j,\;e,\;p}}{K_{m,\;i,\;j,\;e,\;p}\left(1+\sum_{p=1}^{NP_{ent}}\frac{{\left[OS\right]}_{i,\;j,\;e,\;p}}{K_{m,\;i,\;j,\;e,\;p}}\right)}$$

For $e\in {E}_{BiBi}$ and $k\in {K}_{e}$, the reaction rate $r_{BiBi,\;i,\;j,\;e,\;k,\;p}$ is given by:

2 $$r_{BiBi,\;i,\;j,\;e,\;k,\;p}=\frac{k_{f,\;j,\;e}{\left[ENZ\right]}_{j,\;e}{{\left[NSD\right]}_{j,\;k}\left[OS\right]}_{i,\;j,\;e,\;p}}{K_{m,\;i,\;j,\;e,\;p}K_{md,\;j,\;e,\;k}\left[1+\frac{{\left[NSD\right]}_{j,\;k}}{K_{md,\;j,\;e,\;k}}\left(1+\sum_{p=1}^{NP_{ent}}\frac{{\left[OS\right]}_{i,\;j,\;e,\;p}}{K_{m,\;i,\;j,\;e,\;p}}\right)\right]}$$

where $k_{f,\;j,\;e}(\text{min}^{-1})$ is the rate-limiting turnover rate of the reaction catalysed by the ${e}^{th}$ enzyme involved in the ${j}^{th}$ reaction, ${\left[ENZ\right]}_{j,\;e}\left(\mu M\right)$ is the intra-Golgi concentration of the ${e}^{th}$ enzyme catalysing the ${j}^{th}$ reaction, ${\left[NSD\right]}_{j,\;k}\left(\mu M\right)$ is the intra-Golgi concentration of the ${k}^{th}$ NSD utilised in the ${j}^{th}$ reaction, ${\left[OS\right]}_{i,\;j,\;e,\;p}\left(\mu M\right)$ is the ${i}^{th}$ plausible alternative oligosaccharide substrate attached to the ${p}^{th}$ protein entity for the ${e}^{th}$ enzyme involved in the ${j}^{th}$ reaction, $K_{m,\;i,\;j,\;e,\;p}\left(\mu M\right)$ is the dissociation constant of the complex formed between the ${i}^{th}$ oligosaccharide substrate attached to the ${p}^{th}$ protein entity and the ${e}^{th}$ enzyme involved in the ${j}^{th}$ reaction, and $K_{md,\;j,\;e,\;k}\left(\mu M\right)$ is the dissociation constant of the complex formed between the ${k}^{th}$ NSD and the ${e}^{th}$ enzyme involved in the ${j}^{th}$ reaction. $N{P}_{ent}$ stands for the number of protein entities considered.

Finally, this model considered that the linear velocity with which each protein entity transits the Golgi apparatus was inversely proportional to its molecular weight. This consideration was previously introduced by del Val et al. [33], who investigated variations in recombinant protein N-linked glycosylation as a function of the cellular secretory capacity. The production rate of both protein entities was assumed to be equal. Model parameters that were not included in the parameter estimation problem were held constant based on literature values and experimental information from previous kinetic modelling studies in the field [32, 34, 44] (see Supplementary Information). Regarding model simulation, the resulting system of non-linear equations was simulated using MINPACK’s modified Powell hybrid method (*hybr*) as implemented in *SciPy*.

### Parameter estimation

The primary goal of the proposed methodology was to infer the joint distribution of kinetic parameter values (e.g. enzyme levels, dissociation constants) for a kinetic model describing a given GRN to recapitulate experimentally observed glycoprofiles of varying complexity. The Bayesian paradigm provided a natural framework for such a parameter estimation task, particularly the use of an Approximate Bayesian Computation scheme (ABC) [45, 46]. In this work, the popular Sequential Monte Carlo (SMC) variant of ABC was implemented [47–49]. ABC-SMC is a simulation-based framework that approximates the posterior distribution of a parameter through a population of sampled parameter values called particles. This particle population undergoes sequential refinement through multiple generations (i.e. iterations) to improve the approximation of the posterior distribution. This methodology, briefly discussed here according to Toni et al. [49], begins by establishing a population $P$ of size $n$ consisting of particles $\theta$ each assigned a positive weight $w$, formally denoted as $P={\{({w}_{q}, {\theta }_{q})\}}_{q=1}^{n}$ with ${\theta }_{q}\in {\mathbb{R}}^{l}$ of dimension $l$ which corresponds to the number of parameters of interest in $\theta$. Based on the population $P$, a sequence $D$ of weighted distances ${\delta }_{q}$ denoted as $D={\{({w}_{q}, {\delta }_{q})\}}_{q=1}^{n}$ is computed based on a distance function $d$ that compares the model prediction ${s}_{pred}$, as obtained by simulation using ${\theta }_{q}$, with the observed data ${s}_{obs}$. A kernel density estimator (KDE) is then employed to transform this population $P$ into a probability density function (PDF), which serves as the proposal distribution for generating future populations. This proposal distribution introduces perturbations to the new $\theta$, allowing the exploration of the parameter space to adapt and evolve in response to the underlying data structure.

In this work, a multivariate normal KDE was used. The initial population is drawn with equal $w$ from the prior distribution ${r}_{0}$ assigned for each parameter, thus serving as the first proposal distribution. An acceptance threshold $\varepsilon$ is also initialised to reflect the distance to ${s}_{obs}$. This threshold $\varepsilon$ can be a single constant value, a predefined list of values corresponding to each generation or dynamically adjusted after each generation. In this work, $\varepsilon$ was set to the median of the weighted distances $D$ of the previous population. The iterative ABC-SMC procedure stops either after a predetermined number of generations or a minimum acceptance threshold ${\varepsilon }_{min}$ is achieved. As a distance function, the root mean square error (RMSE) metric was used. ABC-SMC was carried out in this work using $p$ *yABC* (version 0.12.13) [50].

The approximation of the posterior distribution of the model parameters of interest was not carried out in a single stage but rather, inspired by previous studies [32–34], a sequential approach was designed and implemented. This approach required the determination of a set of critically important nodes in the GRN, henceforth referred to as *critical nodes* and a set of experimentally observed nodes, called *observed nodes*, which was the list of N-glycan structures measured experimentally in the analysis under investigation. The glycan nomenclature used to describe nodes in the GRN throughout this work was LinearCode [51, 52] and the experimentally observed nodes were characterised by compositional tags based on the Oxford notation (e.g. M5, FA2). Given structural isomorphisms, it should be noted that a single compositional tag could be assigned to multiple LinearCode representations (e.g. FA2G1 may contain a galactose residue at either a1-3 or a1-6 arm). In cases like this, all potential critical nodes were considered in this analysis.

The criterion for selecting critical nodes in GRN considered both the betweenness centrality of nodes and their membership in the dominance frontiers. Betweenness centrality measures the extent to which a node lies on the shortest paths between other nodes in the network, highlighting its role as a crucial intermediary in the flow of information or substrates. Nodes with high betweenness centrality are pivotal in maintaining the connectivity and function of the network. Additionally, in this biosynthetic network, these nodes were expected to be early-appearing, representing important species that would act as substrates for multiple downstream reactions. To ensure comprehensive network coverage and to select nodes beyond early biosynthetic pathways, we incorporated an additional layer of analysis by evaluating the dominance frontier membership of each node. The dominance frontier of a node captures its influence over the connectivity and reachability of other nodes. By selecting nodes with high betweenness centrality and significant dominance frontier memberships, we ensured that our chosen critical nodes are not only central intermediaries but also key structural connectors. This dual criterion, based on percentile thresholds for both metrics, allowed us to determine as critical nodes those that exhibit above-average betweenness centrality and substantial dominance frontier memberships. Given the set of critical nodes, the next goal was to determine the number of parameter estimation stages that should be implemented, and, within each estimation stage, which enzymes and which glycan relative abundances should be included. To achieve this, GlyCompute includes a functionality that automates the process of deriving the sequential parameter estimation strategy (code available in strategy.py module on https://github.com/kf120/GlyCompute_paper) outlined as follows:

- Step 1: Initialisation

First, the strategy ensured inter-conversion between LinearCode and compositional tags using necessary mappings and set up data structures to store data for each stage, as well as to keep track of enzymes already considered. The experimentally observed relative abundances were supplied as input to the algorithm. These observed abundances serve as the target data for the model fitting process, guiding the parameter estimation to find values for the kinetic parameters that best reproduce the experimental results.

- Step 2: Identification of enzyme participation in convergent paths

Iteration over each tag identifying all corresponding critical nodes was carried out. Each stage was defined by a segment of the graph delineated by the source nodes and the critical nodes. Within each stage, all simple paths from the source nodes to the critical nodes were examined and, for each path, all unique enzymes were selected only if they had not been chosen by previous stages. Once a stage is processed, the critical nodes from the previous stage become the source nodes for the subsequent stage.

- Step 3: Assignment of stage-specific relative abundance profiles

To determine the stage-specific relative abundance profiles, all observed nodes preceding the critical nodes within the given stage were identified (termed *preceding observed nodes*). These nodes were assigned to the relative abundances from the experimentally observed glycoprofile. The remaining abundance was assigned to the critical nodes identified in the stage. The rationale was that the enzyme levels and kinetics forming the glycosylation machinery should be capable of capturing experimentally observed glycan abundances and accumulating the remaining abundance to the critical nodes when downstream enzymes were not active.

- Step 4: Identification of enzyme participation in divergent paths

After processing all critical tags, the strategy identified any remaining enzymes not selected in previous stages. For their estimation, the original experimentally observed glycoprofile was used.

Once the strategy was proposed by the algorithm, ABC-SMC was implemented in each stage. To demonstrate a scenario where the user does not have reliable prior information about the model parameters, an uninformative uniform prior was assigned to all model parameters in the stage-wise estimation approach. Once a stage was finished and the posterior distribution of the parameters under investigation was approximated, their values were fixed to the maximum a posteriori (MAP) estimate for the subsequent parameter estimation stages. The relative abundances of the experimentally observed N-glycans for the IgG and HCP glycoprofile were taken from [39].

## Results

In this work, we propose GlyCompute, a computational framework for the streamlined construction of GRNs for N-linked protein glycosylation, followed by the derivation of a kinetic glycosylation model based on known enzyme mechanisms and specificities, and model parameter estimation. To illustrate the framework, we apply it to describe N-linked glycosylation in CHO cell cultures, which have high industrial relevance in biomanufacturing and for which there is an abundance of experimental data. Demonstration focuses on the challenging task of parameterising the kinetic model for simultaneously predicting recombinant IgG and HCP N-linked glycoprofiles in CHO cell culture.

### Subgraph generation using IgG and HCP glycoprofiles

**Fig. 2:**
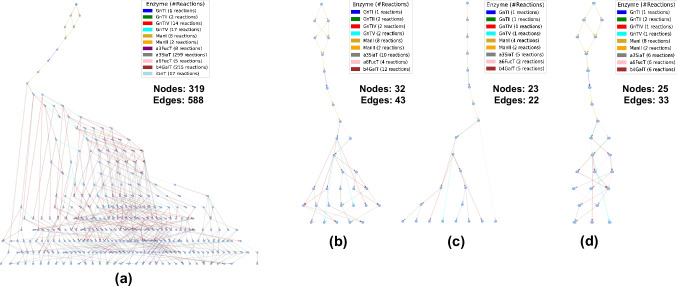
GRNs considered in this study, namely (**a**) template GRN (CHOGlycoNET), (**b**) SPF, (**c**) MFR, and (**d**) GLW

CHOGlycoNET was used as a template GRN. This GRN considered the presence of 11 N-linked glycosylation Golgi-resident enzymes, namely ManI (MAN1A1 and MAN1A2), ManII (MAN2A1 and MAN2A2), GnTI (MGAT1), GnTII (MGAT2), GnTIV (MGAT4A and MGAT4B), GnTV (MGAT5), b4GalT (B4GALT1-7), a3SiaT (ST3Gal3, 4, and 6), a6FucT (FUT8), a3FucT (FUT3-7), and iGnT (B3GNT2). The presence of these enzymes in CHOGlycoNET could lead to the synthesis of elaborate N-glycan structures up to tetra-antennary complex N-glycans with poly-N-acetyllactosamine (poly-LacNAc) extensions. Nonetheless, fitting the model to such elaborate N-glycan structures may not be necessary in cases where the experimentally observed N-linked glycoprofiles do not display such a level of elaboration and/or highly elaborate structures are present in very small relative abundances (e.g. below 1%). From a computational perspective, using the full CHOGlycoNET GRN would lead to a mathematical model described by a large system of non-linear equations, thus requiring large simulation times to achieve convergence. Taking the above into consideration, CHOGlycoNET was pruned to include all experimentally observed N-glycan structures found at a relative abundance of over 1% across all IgG and HCP protein entities [39]. Such N-glycan structures include high-mannose N-glycans, core-fucosylated and fucosylated bi-antennary complex N-glycans with/without terminal sialic acid, and core-fucosylated tri-antennary N-glycans. The implementation of SPF and MFR led to a significant reduction in both node and edge counts relative to the template GRN (Fig. 2). The smallest GRN was generated by MFR, which led to a reduction of 93% with respect to node count and 97% to edge count. The GLW approach also led to a small GRN with 25 nodes and 33 edges (Fig. 2).

The comparison of the GRNs represented by the SPF, MFR, and GLW graphs revealed varying degrees of structural similarity and dissimilarity (Table 1). The SPF and MFR graphs exhibited significant overlap, sharing 23 nodes and 22 edges. Despite this overlap, the graphs were not isomorphic, indicating distinct graph topologies. This was further evidenced by the graph edit distance (GED) between SPF and MFR, which was 70, highlighting the large number of graph operations required to transform SPF into a graph isomorphic to MFR. Notably, the MFR graph was a subgraph of the SPF graph, suggesting that the SPF graph contained all N-glycan biosynthetic pathways present in the MFR graph plus additional pathways. In contrast, the comparison between the SPF and GLW graphs showed lesser overlap, with only 18 nodes and 13 edges in common. These graphs were also not isomorphic, with a higher GED of 83, indicating more pronounced topological differences compared to the SPF-MFR pair. Among the graph pairs, the MFR and GLW graphs showed the least similarity, sharing only 13 nodes and 8 edges. These graphs were not isomorphic and had the smallest observed GED of 57. To enable the assessment of the GED magnitude across all mGRN pairs, each GED was normalised by the average edge count of the respective pair. The consistent finding of a normalised GED by edges close to 2 across all comparisons indicated that, on average, each edge would require approximately two modifications to align the graphs. This underscored the significant structural changes required to transform one GRN into another, reflecting substantial topological differences among the SPF, MFR, and GLW graphs. This was favourable since the topologically different mGRNs could likely impact the goodness-of-fit between the predictions of their respective kinetic models and the experimentally observed glycoprofiles. It is worth noting that all three mGRNs omit glycosyltransferases that were responsible for more elaborate processing of the N-glycan structures, namely a3FucT and iGnT, which catalyse the reactions producing Lewis X (Le^X^) antigen epitopes and poly-LacNAc extensions, respectively.

**Table 1:** Pairwise graph comparisons between SPF, MFR, and GLW. GED was normalised by the average edge count of the graph pair compared

Graph pair	Node overlap	Edge overlap	Isomorphic	GED	GED (normalised)	Subgraph (graph 1 of graph 2)	Subgraph (graph 2 of graph 1)
SPF-MFR	23	22	×	70	2.15	×	✓
SPF-GLW	18	13	×	83	2.18	×	×
MFR-GLW	13	8	×	57	2.07	×	×

### Parameterisation using the sequential parameter estimation strategy

**Fig. 3:**
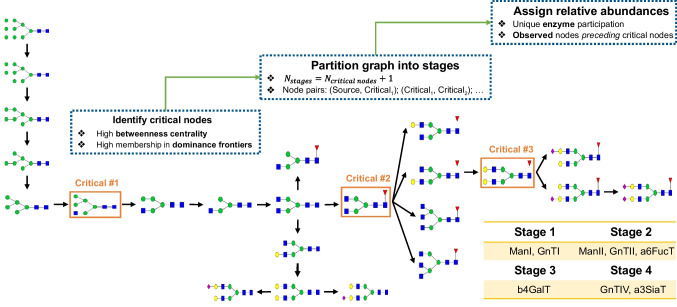
Schematic of the automated sequential parameter estimation strategy developed in this work and applied to the mGRN_MFR_. Orange rectangles indicate critical nodes identified based on above-average (i.e. above 50th percentile) values of node betweenness centrality and membership in the dominance frontiers of all nodes. The table shows the four stages determined by the automated strategy, along with the corresponding enzymes whose kinetic parameters need to be estimated

To enable the streamlined analysis and parameterisation of kinetic models of protein N-linked glycosylation, GlyCompute incorporates a sequential parameter estimation strategy based on ABC-SMC. As a first step, the critical nodes were identified based on high betweenness centrality and membership to dominance frontier sets, as explained in the “Parameter estimation” section, using each respective mGRN. In all cases, these corresponded to the compositional tags M5A1, FA2, and FA2G2. This selection was in line with domain knowledge since M5A1 is a key node in the initial, convergent pathway starting from M9 and proceeding via the action of ManI and GnTI that acts as a crucial substrate to further processing and maturation of N-glycans (Fig. 2). The selection of FA2 as a critical node was also justified, as it represents a core-fucosylated bi-antennary N-glycan that can be further elaborated by multiple enzymes, including b4GalT, GnTIV, and GnTV. The action of b4GalT on FA2 catalyses the synthesis of the final critical tag, namely FA2G2. While FA2G2 is part of divergent pathways, it was deemed critical in the overall coverage of the different mGRNs, as it is the substrate of a3SiaT for the synthesis of sialylated N-glycans found in the HCP glycoprofile. The choice of these three N-glycan structures as critical nodes led to a four-stage parameter estimation process, which was implemented separately for the IgG and HCP glycoprofiles (Fig. 3). Since a single GRN was used to describe the biosynthesis of N-glycans found on both protein entities, the number of stages was the same and only the actual relative abundance values suggested by the sequential parameter estimation algorithm differed.

The first stage suggested the estimation of parameters related to ManI and GnTI by setting the relative abundance of all observed high-mannose structures that preceded M5A1 to their original experimental values and reassigning all remaining relative abundance to M5A1. Then, the second stage suggested the simultaneous estimation of parameters related to ManII, GnTII, and a6FucT by setting the relative abundance to all observed high-mannose N-glycans and all bi-antennary afucosylated and agalactosylated N-glycans that preceded FA2 to their original experimental values and reassigning the relative abundance of all remaining glycans to FA2. The third stage suggested the estimation of parameters related only to b4GalT by setting the relative abundance of all observed high-mannose and bi-antennary fucosylated agalactosylated N-glycans that preceded FA2G2 to their original experimental values and reassigning all remaining relative abundance values to FA2G2. Finally, the fourth stage suggested the estimation of the parameters related to the remaining enzymes GnTIV, GnTV, and a3SiaT based on the original experimentally observed relative abundances of the glycoprofile of interest.

**Fig. 4:**
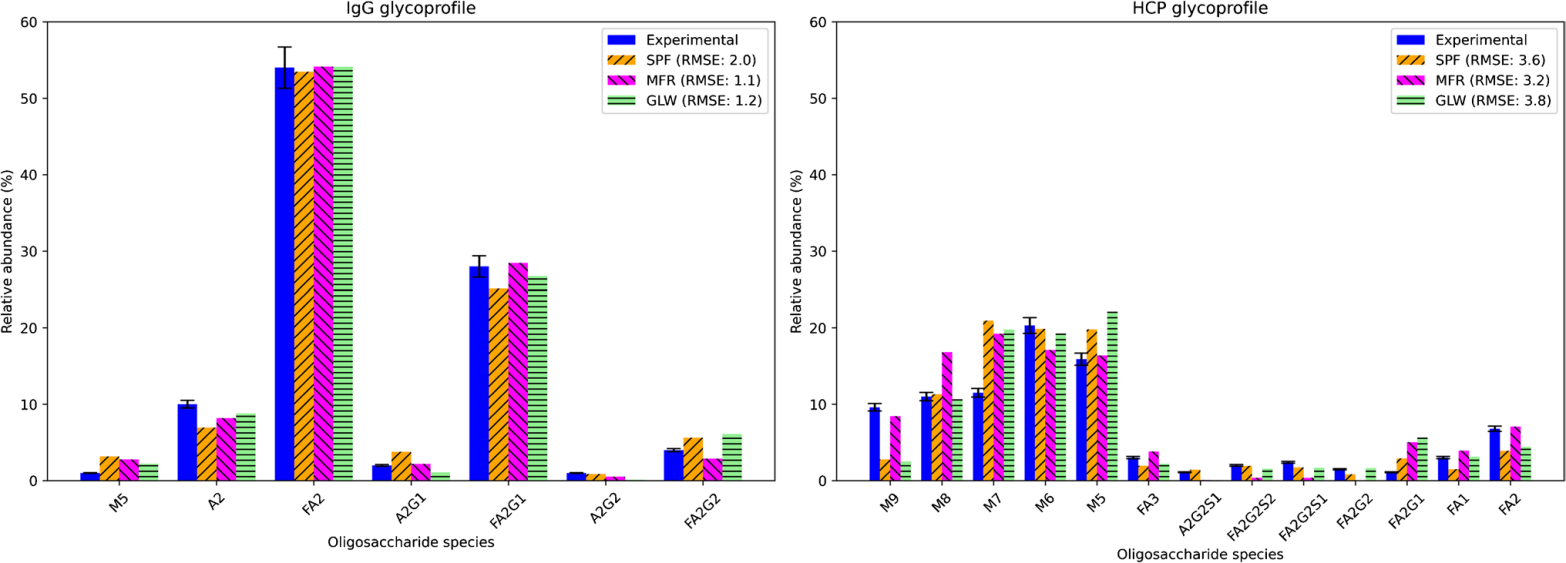
Goodness-of-fit between model predictions across all mGRNs and the experimental observations for the IgG and HCP glycoprofiles

The kinetic parameters estimated in this study were the mass fractions of M8:M9 entering the Golgi apparatus, total enzyme protein levels in the Golgi ($[ENZ]$), and the dissociation constants associated with HCP ($K_{m,\;HCP}$). The prior distributions were set to $U(0.05, 0.95$), $U(0.001, 35$), and $U(10, 10000)$, respectively. The population size $P$ was set to 200 and the maximum number of generations to 25, values which were found to provide a satisfactory balance between a refined approximation of the posterior distributions of the parameters and computational overhead. All parameter estimation activities were run in parallel using 64 cores. Figure 4 depicts the goodness-of-fit between the model predictions and the experimental observations for all three mGRNs.

Overall, the kinetic models based on the three mGRNs all achieved a satisfactory agreement with the experimental observations capturing the distinct trends found in both glycoprofiles. In particular, the model in all three cases was able to reproduce, both qualitatively and quantitatively, the distinct trends in the IgG and HCP glycoprofiles. These corresponded to the high abundance of core-fucosylated bi-antennary complex N-glycans in the IgG glycoprofile, with FA2 represented at over 50% relative abundance and the presence of the highly abundant high-mannose N-glycans (i.e. M9, M8, M7, M6, M5) in the HCP glycoprofile, a phenotype that was not observed in IgG. The mGRN_MFR_ kinetic model showed the best agreement with a total RMSE of 4.3, marginally surpassing the kinetic models based on mGRN_SPF_ and mGRN_GLW_, which had RMSE scores of 5.6 and 5.0, respectively. For the IgG glycoprofile, the mGRN_MFR_ model fit high-abundance species such as FA2 and A2G1 well. Similarly, for the HCP glycoprofile, the mGRN_MFR_ model’s predictions for high-abundance species such as M9 and M8 were more closely aligned with the experimental values, and it maintained good performance for low-abundance species. The mGRN_SPF_ and mGRN_GLW_ models performed quite similarly, providing a reasonably accurate fit overall.

Given the comparable predictive performance of all three model configurations despite the topological differences of the underlying mGRNs, as highlighted in the “Subgraph generation using IgG and HCP glycoprofiles” section, the results herein indicate that the choice of GRN topology may not profoundly affect parameterisation and goodness-of-fit. This is an interesting outcome suggesting that, as long as the underlying GRN can capture key biosynthetic pathways of protein N-linked glycosylation relevant to the experimentally observed glycoprofiles, the in-sample prediction accuracy of the kinetic model rather lies on the parameterisation strategy implemented.

Table 2 summarises the MAP estimates and 95% credible intervals (CrI) of all estimated parameters. Regarding the majority of the total enzyme concentrations, their MAP estimates were fairly close across different mGRN-based kinetic models, with overlapping 95% CrI. Significant overlap between the 95% CrI across the different mGRN-based kinetic models was also observed in the estimation of the HCP-related dissociation constants; however, their CrI were wide, spanning one order of magnitude, regardless of model configuration. Interestingly, their MAP estimates were consistently higher than the IgG-related dissociation constants (see Supplementary Information). This indicates that, to match the experimentally observed glycoprofiles, the affinity of the Golgi-resident enzymes towards the HCP N-glycans had to be much lower than the one towards the IgG N-glycans. This behaviour could be explained based on the distinct trends found in the two experimental glycoprofiles. The lighter protein entity (in terms of molecular weight) HCP would have to transit through the Golgi at a higher linear velocity, and for a given set of enzyme levels, a lower degree of processing and maturation could only be explained in this context by a smaller affinity towards its glycans. The wide CrI in the case of the HCP-related dissociation constants, as opposed to the comparatively narrower CrI in the case of total enzyme levels, indicated that significant changes in the values of the former do not markedly influence model outputs. Such an insight would typically be gained via global sensitivity analysis [53], a step that typically precedes parameter estimation. Albeit powerful, implementing variance-based global sensitivity analysis in this scenario would have increased the computational overhead of the overall framework and, at the same time, would return a large set of highly influential parameters due to high-order interactions induced by the non-linear correlations between the parameters under investigation (data not shown). Consequently, the implemented ABC-SMC strategy still provided insights towards model reduction, while simultaneously integrating the process of approximating the posterior distribution of the parameters.

**Table 2:** Results of sequential parameter estimation approach based on ABC-SMC

Parameter (units)	MAP estimate (95% CrI)		
	SPF	MFR	GLW
$M{9}_{prop, IgG} (-)$	0.58 (0.07, 0.92)	0.19 (0.06, 0.91)	0.44 (0.08, 0.93)
$M{9}_{prop, HCP} (-)$	0.85 (0.47, 0.95)	0.74 (0.40, 0.92)	0.90 (0.70, 0.95)
${\left[ENZ\right]}_{ManI} (\mu\text{M})$	0.25 (0.15, 0.35)	0.29 (0.19, 0.42)	0.41 (0.23, 0.43)
${\left[ENZ\right]}_{ManII} (\mu\text{M})$	15.75 (4.63, 34.13)	27.27 (3.46, 33.90)	20.96 (2.61, 34.16)
${\left[ENZ\right]}_{GnTI} (\mu\text{M})$	0.28 (0.21, 0.5)	0.29 (0.23, 0.43)	0.26 (0.21, 0.39)
${\left[ENZ\right]}_{GnTII} (\mu\text{M})$	0.77 (0.30, 1.62)	14.42 (7.69, 34.29)	0.53 (0.28, 1.09)
${\left[ENZ\right]}_{GnTIV} (\mu\text{M})$	0.09 (0.004, 0.48)	0.04 (0.02, 0.08)	0.02 (0.01, 0.09)
${\left[ENZ\right]}_{GnTV} (\mu\text{M})$	0.01 (0.002, 0.04)	0.52 (0.09, 0.76)	0.39 (0.17, 0.83)
${\left[ENZ\right]}_{a6FucT} (\mu\text{M})$	0.09 (0.09, 0.09)	0.09 (0.08, 0.09)	0.08 (0.07, 0.08)
${\left[ENZ\right]}_{b4GalT} (\mu\text{M})$	0.02 (0.02, 0.02)	0.02 (0.02, 0.02)	0.02 (0.02, 0.02)
${\left[ENZ\right]}_{a3SiaT} (\mu\text{M})$	0.33 (0.12, 0.80)	26.15 (6.51, 33.74)	0.26 (0.08, 0.60)
$K_{m,\;HCP,\;ManI}(\mu\text{M})$	5584 (3480, 9596)	6079 (3576, 9639)	8349 (5232, 9852)
$K_{m,\;HCP,\;ManII}(\mu\text{M})$	2466 (236, 9624)	2669 (302, 9828)	2461 (268, 9425)
$K_{m,\;HCP,\;GnTI}(\mu\text{M})$	8885 (4797, 9954)	8586 (6132, 9897)	9215 (6408, 9877)
$K_{m,\;HCP,\;GnTII}(\mu\text{M})$	8172 (4057, 9584)	8625 (2743, 9722)	8778 (3025, 9898)
$K_{m,\;HCP,\;GnTIV}(\mu\text{M})$	8260 (1642, 9961)	8662 (4329, 9820)	8304 (2464, 9828)
$K_{m,\;HCP,\;GnTV}(\mu\text{M})$	3777 (1345, 9849)	6910 (3824, 9859)	6817 (2071, 9775)
$K_{m,\;HCP,\;a6FucT}(\mu\text{M})$	306 (211, 389)	48 (12, 117)	205 (95, 315)
$K_{m,\;HCP,\;b4GalT}(\mu\text{M})$	1015 (958, 1078)	2605 (2479, 2642)	1096 (1061, 1178)
$K_{m,\;HCP,\;a3SiaT}(\mu\text{M})$	7889 (1897, 9734)	583 (74, 3690)	7420 (1904, 9935)

**Fig. 5:**
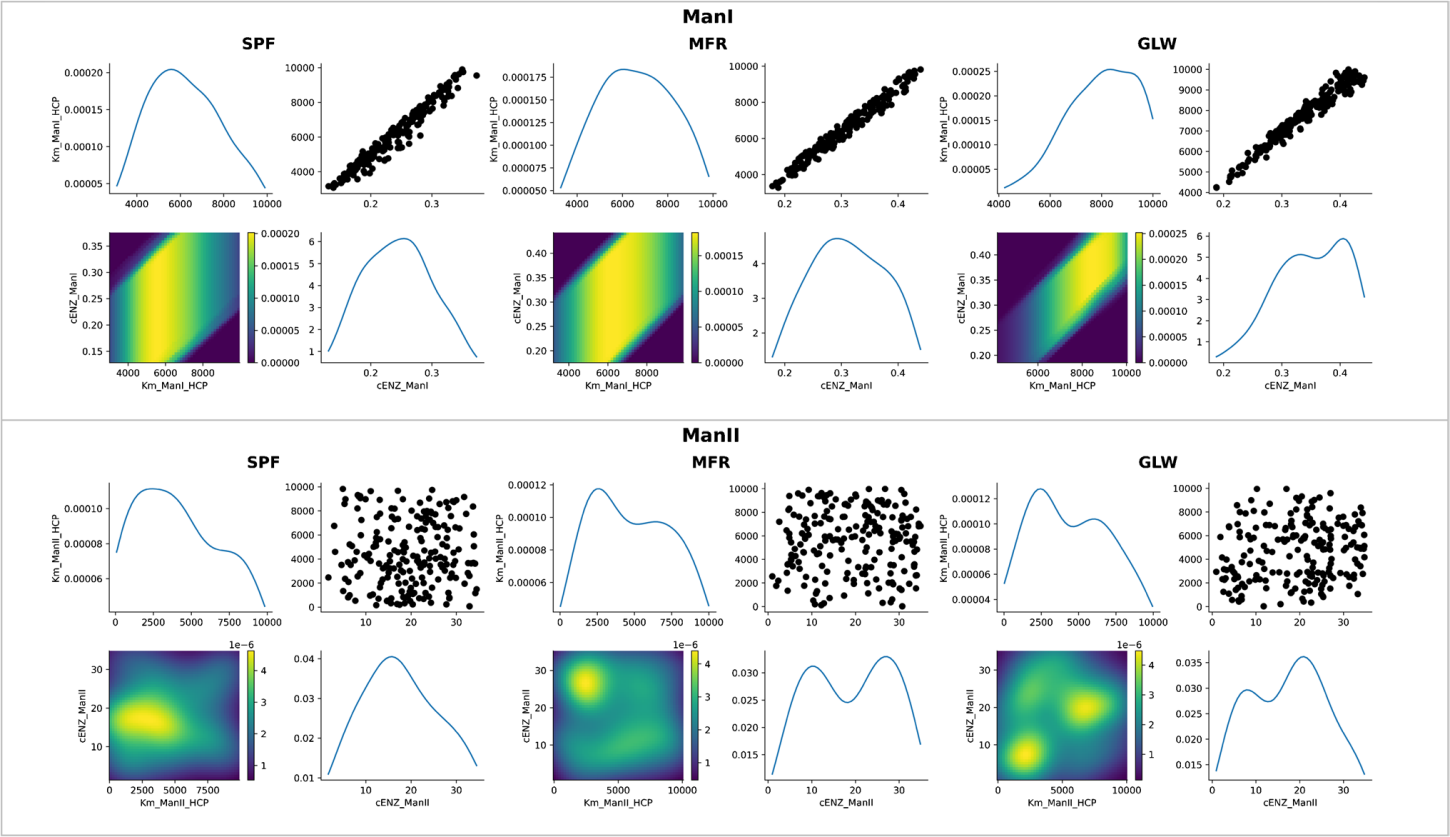
Approximate posterior distributions of kinetic parameters across mGRNs per glycosidase

**Fig. 6:**
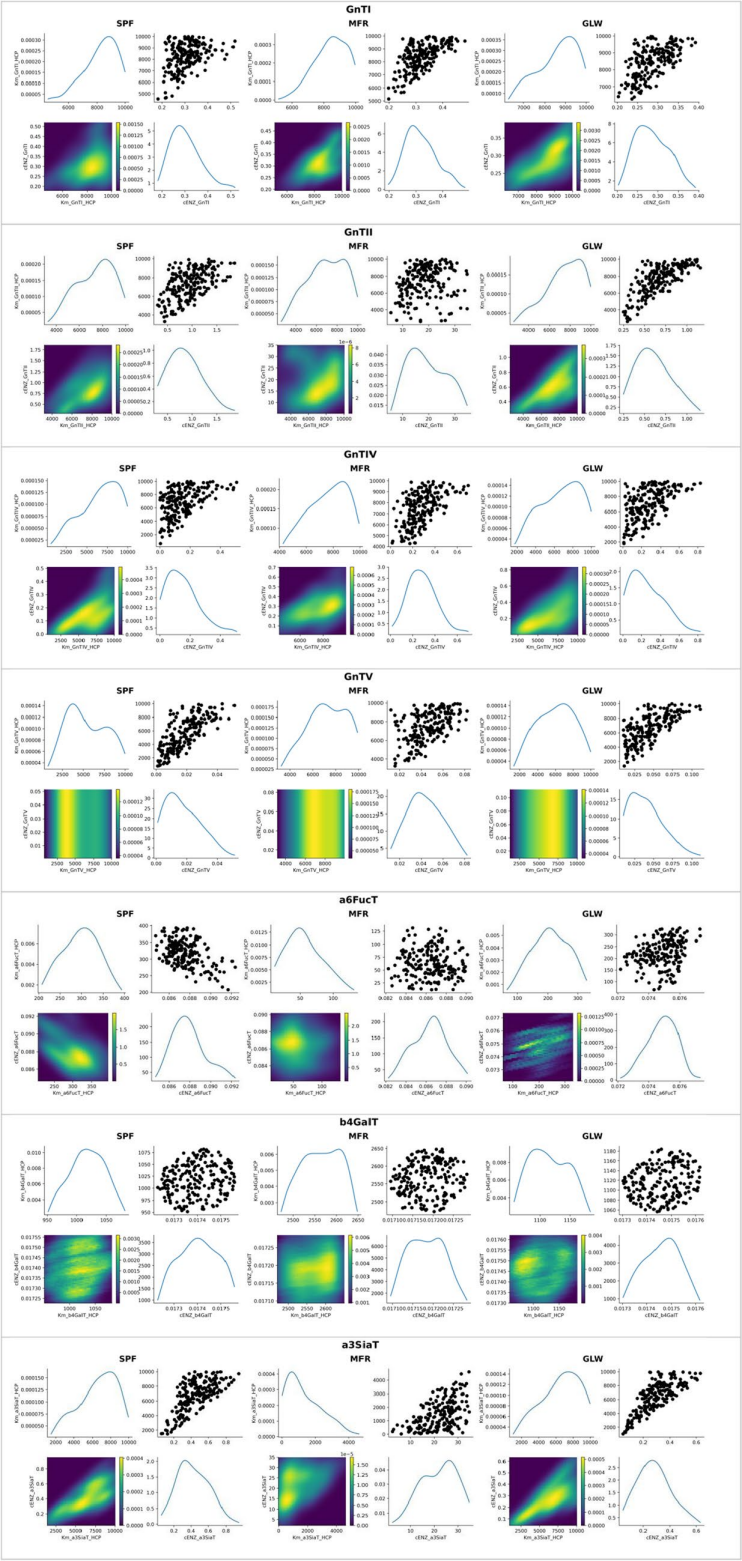
Approximate posterior distributions of kinetic parameters across mGRNs per glycosyltransferase

The approximate posterior distributions are shown in Fig. 5 and Fig. 6. The two-dimensional approximate posterior joint distributions per enzyme revealed the associations between the total enzyme levels and the HCP-related dissociation constants. In particular, a positive linear correlation was observed between the total enzyme concentration of ManI and its HCP-related dissociation constant across all model configurations, with several probable pairs of values around the MAP estimates of the two parameters. More complex, non-linear associations can be discerned for the enzymes responsible for branching, including GnTI, GnTII, and GnTIV, as well as for a3SiaT. In the case of the branching enzymes, the KDE was skewed towards high values of the dissociation constants and low values of enzyme concentrations across all model configurations, a trend that was also observed in the case of a3SiaT for the mGRN_SPF_ and mGRN_GLW_ kinetic models, but not for mGRN_MFR_, where the trend was reversed. Finally, no clear associations could be seen for the approximate posteriors of a6FucT and b4GalT. These observed associations could be driven by several factors inherent to the kinetic mechanisms and the structure of the glycosylation pathways. The positive linear correlation between the total enzyme concentration of ManI and its HCP-related dissociation constant might reflect the substrate saturation effect characteristic of Michaelis–Menten kinetics, where higher enzyme concentrations necessitate higher dissociation constants to maintain a balanced reaction rate. For the branching enzymes such as GnTI, GnTII, and GnTIV, the non-linear associations might be indicative of more complex kinetic mechanisms, such as Bi-Bi sequential kinetics, where the interplay between multiple substrates and enzyme conformations creates a more intricate relationship between enzyme levels and dissociation constants. The skewed distributions for these enzymes suggest that in conditions of high dissociation constants, the enzyme concentrations are kept low to potentially avoid excessive branching that could lead to tetra-antennary N-glycans, not present in the experimentally observed glycoprofiles. The lack of clear associations for a6FucT and b4GalT indicates a potential robustness in their kinetic parameters, where narrow CrI suggest a tightly regulated enzymatic rate that is less sensitive to fluctuations in enzyme concentration and dissociation constants. The joint distributions provided a richer understanding of parameter interactions than point estimates alone, highlighting the benefit of implementing ABC-SMC compared to classical MLE approaches, which only produce point estimates and lack the additional information offered by distributions.

## Discussion

Bioinformatics efforts are underway to incorporate glycosylation into the central dogma of biology due to its ubiquitous presence in cellular communication and molecular interactions [7, 54]. The recent surge in algorithmic advancements and approaches hosted in web-based platforms and/or open-source libraries has given experimental biologists unprecedented access to computational tools to analyse their data. Further attempts to design modular methodologies to interrogate glycomic data could aid the quantitative analysis of glycosylation pathways. The present work proposes GlyCompute, a methodological framework demonstrating a streamlined approach for the assembly, simulation, and parameterisation of kinetic models of protein N-linked glycosylation. It is noted that the current version of GlyCompute does not explicitly address structural ambiguities in glycans, such as linkage uncertainties or monosaccharide ambiguities; rather, it considers GRNs generated based on known enzymatic specificities. We envision that GlyCompute, together with other community tools for computer-aided glycosylation analysis will support researchers to gain quantitative insights about the effect of enzyme kinetics and perturbations thereof on experimentally observed glycoprofiles. To achieve this, GlyCompute incorporates an automated sequential parameter estimation approach based on the Bayesian paradigm. Its application is illustrated on the parameterisation of a kinetic model of protein N-linked glycosylation for the simultaneous fitting to the experimentally observed glycoprofiles on two different protein entities, a recombinant IgG and HCP, produced by the same CHO cell culture. This case study was challenging since the parameter estimation approach would have to approximate the posterior distributions of kinetic parameters (i.e. enzyme protein levels, dissociation constants) that could capture distinct trends in both glycoprofiles simultaneously. As seen in the “Parameterisation using the sequential parameter estimation strategy” section, the experimentally observed IgG glycoprofile primarily comprised bi-antennary asialylated complex N-glycans, whereas the experimentally observed HCP glycoprofile predominantly consisted of high-mannose N-glycans. This indicated that the degree of N-glycan maturation for HCP was much less pronounced, which could be attributed to an overall small residence time in the Golgi apparatus due to the smaller molecular weight compared to IgG, reduced affinity of glycosyltransferases to the N-glycans found on HCP, and/or low glycosyltransferase concentration levels. At the same time, the actual size of the GRN representing the biosynthetic pathways in the Golgi cannot be experimentally validated in vivo, thus introducing further uncertainty in the data-generating process. Nonetheless, these hypotheses could be explicitly tested quantitatively using the kinetic model. Indeed, the parameter estimation approach indicated that regardless of the subgraph representation of the GRN that was used, lower affinity towards HCP N-glycans, as shown by the large estimated $K_{m,\;HCP}$ values, could explain the experimentally observed glycoprofiles with satisfactory accuracy. The hypothesis of low glycosyltransferase concentration levels could not individually explain both experimentally observed glycoprofiles, as core-fucosylated bi-antennary N-glycans, such as FA2, were found at high abundance in the one glycoprofile (i.e. IgG). This suggested that the overall glycosylation machinery had the required resources (i.e. enzyme levels) to produce these structures.

It should be acknowledged that GlyCompute remains to be tested more broadly before it can be robustly integrated in computer-aided glycosylation analysis. In this study, three subgraph generation methods were considered, two pruning methods implemented on a template GRN (i.e. CHOGlycoNET) and the network construction method in Glycowork that did not require a template GRN but utilised a novel evolutionary pruning approach. CHOGlycoNET was generated using GLYMMER™ and manual curation but it is not the only option to obtain a template GRN. Users can also use the web-based GPP tool to generate GRNs based on custom enzyme reaction rules and further prune the resulting GRN or not. GlyCompare is another interesting option; it is an open-source tool that can be used to decompose experimentally observed glycoprofiles to a minimal set of intermediate substructures in order to enable an explicit comparison between glycoprofiles of differing complexity. GlyCompare can return a network representation integrating the minimal set of intermediate substructures, which thus, similarly to the network construction functionality in Glycowork, does not require further pruning. Manually constructed GRNs can also be used within the proposed framework, provided that the GRN is expressed as a DAG where the nodes represent the glycan species, with node attributes including a LinearCode description of the structures, and the edges represent the reactions catalysed by the glycoenzymes, with enzyme attributes denoting their names. A limitation of the current algorithmic approach is its dependency on accurately identifying critical nodes. It remains to be confirmed that this approach can capture the full complexity of the glycosylation pathways if the network topology changes significantly or if there are missing interactions.

Future work will focus on implementing this framework as a tool to gain quantitative insights into changes in the glycosylation machinery that could explain aberrant N-linked glycoprofiles from diseased tissues. While GlyCompute currently focuses on N-linked glycosylation, there is potential to expand its scope to other glycan classes, such as O-glycans or glycolipids. This would require several key adaptations. Firstly, detailed information on the kinetic mechanisms and specificities of glycosyltransferases involved in these classes would be necessary. The network analysis tools would then need to be modified to accommodate the unique biosynthetic pathways of each glycan class, potentially requiring adjustments to the network construction and pruning rules. Development or adaptation of template glycosylation reaction networks (GRNs) for these new classes, akin to CHOGlycoNET for N-glycans, would be beneficial, though this might involve extensive manual curation and validation. To achieve this, the framework would need to account for the broader range of substrate specificities observed in other glycan classes, particularly the diverse core structures of O-glycans. Finally, experimental data for model validation across these different glycan classes would be required to ensure the expanded framework’s accuracy and reliability. Expanding GlyCompute to encompass a broader range of glycan classes could provide valuable insights into the interplay between different glycosylation pathways and their collective impact on cellular function and disease states.

## Supplementary Information

Below is the link to the electronic supplementary material.

Supplementary file1 (DOCX 112 KB)

## Data Availability

The source code necessary for reproducing the results presented in this paper is openly available on GitHub at https://github.com/kf120/GlyCompute_paper.
