# Supplementary material for: GlyCompute: towards the automated analysis of protein N-linked glycosylation kinetics via an open-source computational framework
Source: Anal Bioanal Chem. 2024 Sep 26;417(5):957–72. doi: 10.1007/s00216-024-05522-3 (PMC11782420; doi:10.1007/s00216-024-05522-3)
Supplement: Supplementary file 1 — Supplementary file1 (DOCX 112 KB) [file 216_2024_5522_MOESM1_ESM.docx]

**Supplementary Information**

**GlyCompute: Towards the automated analysis of protein N-linked glycosylation kinetics via an open-source computational framework**

**Authors**

Konstantinos Flevaris^a, *^, Pavlos Kotidis^a^^[[1]](#footnote-1)^, Cleo Kontoravdi^a, *^

**Affiliations**

^a^ Department of Chemical Engineering, Imperial, London SW7 2AZ, United Kingdom

Research Article submitted to: Analytical and Bioanalytical Chemistry

________________________________________

* Authors to whom **correspondence** should be addressed.

E-mail address: [k.flevaris21@imperial.ac.uk](mailto:k.flevaris21@imperial.ac.uk)

E-mail address: [cleo.kontoravdi@imperial.ac.uk](mailto:cleo.kontoravdi98@imperial.ac.uk)

**Table S1**: Experimentally observed N-glycan structures considered in the present study

| Oxford Nomenclature | SNFG | GlyTouCan ID |
| --- | --- | --- |
| M9 | 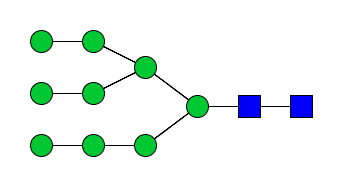 | G56202TA |
| M8 | 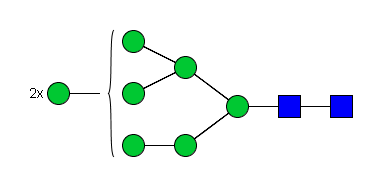 | G59513YA / G63035DM |
| M7 | 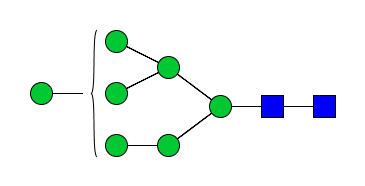 | G83600PQ / G50507PS |
| M6 | 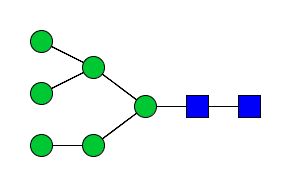 | G48442VP |
| M5 | 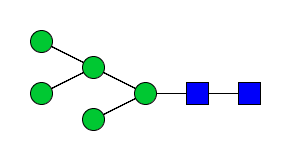 | G03652TR |
| A2 | 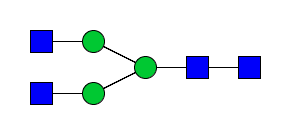 | G88876JQ |
| A2G1 | 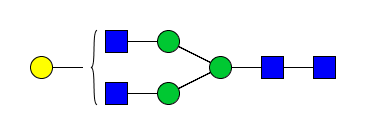 | G36859SD / G44754DF |
| A2G2 | 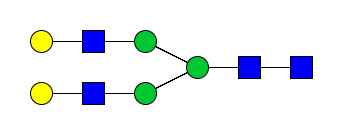 | G66741YQ |
| A2G2S1 | 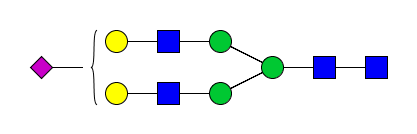 | G01670UQ / G37591JC |
| A2G2S2 | 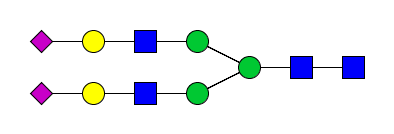 | G73866ZM |
| FA1 | 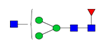 | G14576KZ / G69987TD |
| FA2 | 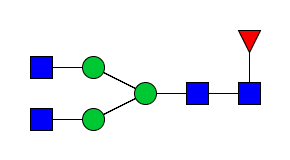 | G65984FE |
| FA2G1 | 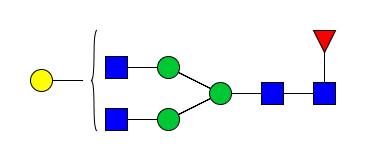 | G29024OJ / G53582JE |
| FA2G2 | 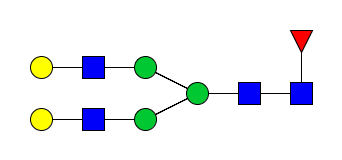 | G00998NI |
| FA2G1S1 | 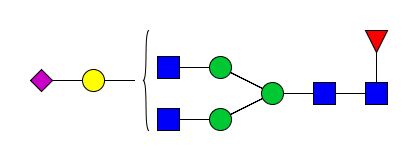 | G71782CJ / G19447ZX |
| FA2G2S1 | 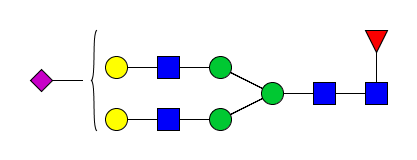 | G01361CV / G88242BL |
| FA2G2S2 | 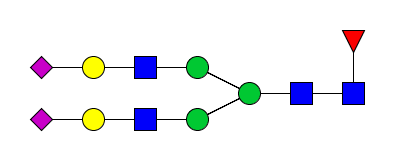 | G72954XC |

**Table S2**: Literature values for model parameters

| Parameter [Units] | Description | Value | Source |
| --- | --- | --- | --- |
| $\boldsymbol{M}\boldsymbol{W}_{\boldsymbol{IgG}}\boldsymbol{[g mo}\boldsymbol{l}^{\boldsymbol{-1}}\boldsymbol{]}$ | Molecular weight of IgG | 150,000 | [1] |
| $\boldsymbol{M}\boldsymbol{W}_{\boldsymbol{HCP}}\boldsymbol{[g mo}\boldsymbol{l}^{\boldsymbol{-1}}\boldsymbol{]}$ | Molecular weight of HCP | 46,167 | [2] |
| $\boldsymbol{G}\boldsymbol{S}_{\boldsymbol{HCP}}\boldsymbol{[-]}$ | Glycosylation sites on HCP | 0.0809 | [2] |
| $\boldsymbol{k}_{\boldsymbol{f, ManI}}\boldsymbol{[}\boldsymbol{min}^{\boldsymbol{-1}}\boldsymbol{]}$ | Turnover rate for ManI | 888 | [1] |
| $\boldsymbol{k}_{\boldsymbol{f, ManII}}\boldsymbol{[}\boldsymbol{min}^{\boldsymbol{-1}}\boldsymbol{]}$ | Turnover rate for ManII | 1924 | [1] |
| $\boldsymbol{k}_{\boldsymbol{f, GnTI}}\boldsymbol{[}\boldsymbol{min}^{\boldsymbol{-1}}\boldsymbol{]}$ | Turnover rate for GnTI | 1022 | [1] |
| $\boldsymbol{k}_{\boldsymbol{f, GnTII}}\boldsymbol{[}\boldsymbol{min}^{\boldsymbol{-1}}\boldsymbol{]}$ | Turnover rate for GnTII | 1406 | [1] |
| $\boldsymbol{k}_{\boldsymbol{f, GnTIV}}\boldsymbol{[}\boldsymbol{min}^{\boldsymbol{-1}}\boldsymbol{]}$ | Turnover rate for GnTIV | 187 | [3] |
| $\boldsymbol{k}_{\boldsymbol{f, GnTV}}\boldsymbol{[}\boldsymbol{min}^{\boldsymbol{-1}}\boldsymbol{]}$ | Turnover rate for GnTV | 1410 | [3] |
| $\boldsymbol{k}_{\boldsymbol{f, a}\boldsymbol{6}\boldsymbol{FucT}}\boldsymbol{[}\boldsymbol{min}^{\boldsymbol{-1}}\boldsymbol{]}$ | Turnover rate for a6FucT | 291 | [1] |
| $\boldsymbol{k}_{\boldsymbol{f, b}\boldsymbol{4}\boldsymbol{GalT}}\boldsymbol{[}\boldsymbol{min}^{\boldsymbol{-1}}\boldsymbol{]}$ | Turnover rate for b4GalT | 872 | [1] |
| $\boldsymbol{k}_{\boldsymbol{f, a}\boldsymbol{3}\boldsymbol{SiaT}}\boldsymbol{[}\boldsymbol{min}^{\boldsymbol{-1}}\boldsymbol{]}$ | Turnover rate for a3SiaT | 491 | [1] |
| $\boldsymbol{K}_{\boldsymbol{m, IgG,ManI}} \left[ \boldsymbol{uM} \right]$ | Dissociation constant of IgG OS from ManI | 61 | [1] |
| $\boldsymbol{K}_{\boldsymbol{m, IgG,ManII}} \left[ \boldsymbol{uM} \right]$ | Dissociation constant of IgG OS from ManII | 100 | [1] |
| $\boldsymbol{K}_{\boldsymbol{m, IgG,GnTI}} \left[ \boldsymbol{uM} \right]$ | Dissociation constant of IgG OS from GnTI | 260 | [1] |
| $\boldsymbol{K}_{\boldsymbol{m, IgG,GnTII}} \left[ \boldsymbol{uM} \right]$ | Dissociation constant of IgG OS from GnTII | 190 | [1] |
| $\boldsymbol{K}_{\boldsymbol{m, IgG,a}\boldsymbol{6}\boldsymbol{FucT}} \left[ \boldsymbol{uM} \right]$ | Dissociation constant of IgG OS from a6FucT | 25 | [1] |
| $\boldsymbol{K}_{\boldsymbol{m, IgG,b}\boldsymbol{4}\boldsymbol{GalT}} \left[ \boldsymbol{uM} \right]$ | Dissociation constant of IgG OS from b4GalT | 430 | [1] |
| $\boldsymbol{K}_{\boldsymbol{md,GnTI}} \left[ \boldsymbol{uM} \right]$ | Dissociation constant of UDPGlcNAc from GnTI | 170 | [1] |
| $\boldsymbol{K}_{\boldsymbol{md,GnTII}} \left[ \boldsymbol{uM} \right]$ | Dissociation constant of UDPGlcNAc from GnTII | 960 | [1] |
| $\boldsymbol{K}_{\boldsymbol{md,GnTIV}} \left[ \boldsymbol{uM} \right]$ | Dissociation constant of UDPGlcNAc from GnTIV | 8300 | [4] |
| $\boldsymbol{K}_{\boldsymbol{md,GnTV}} \left[ \boldsymbol{uM} \right]$ | Dissociation constant of UDPGlcNAc from GnTV | 5390 | [4] |
| $\boldsymbol{K}_{\boldsymbol{md,a}\boldsymbol{6}\boldsymbol{FucT}} \left[ \boldsymbol{uM} \right]$ | Dissociation constant of GDPFuc from a6FucT | 46 | [1] |
| $\boldsymbol{K}_{\boldsymbol{md,b}\boldsymbol{4}\boldsymbol{GalT}} \left[ \boldsymbol{uM} \right]$ | Dissociation constant of UDPGal from b4GalT | 65 | [1] |
| $\boldsymbol{K}_{\boldsymbol{md,a}\boldsymbol{3}\boldsymbol{SiaT}} \left[ \boldsymbol{uM} \right]$ | Dissociation constant of CMPNeuAc from a3SiaT | 57 | [4] |

# **References**

1. Jimenez del Val I, Nagy JM, Kontoravdi C (2011) A dynamic mathematical model for monoclonal antibody N-linked glycosylation and nucleotide sugar donor transport within a maturing Golgi apparatus. Biotechnol Prog 27:1730–1743. https://doi.org/10.1002/btpr.688

2. Del Val IJ, Polizzi KM, Kontoravdi C (2016) A theoretical estimate for nucleotide sugar demand towards Chinese Hamster Ovary cellular glycosylation. Sci Rep 6:1–15. https://doi.org/10.1038/srep28547

3. Krambeck FJ, Betenbaugh MJ (2005) A mathematical model of N-linked glycosylation. Biotechnol Bioeng 92:711–728. https://doi.org/10.1002/bit.20645

4. Krambeck FJ, Bennun S V., Narang S, Choi S, Yarema KJ, Betenbaugh MJ (2009) A mathematical model to derive N-glycan structures and cellular enzyme activities from mass spectrometric data. Glycobiology 19:1163–1175. https://doi.org/10.1093/glycob/cwp081

1. Present address: Biopharm Process Research, GSK, Stevenage, United Kingdom [↑](#footnote-ref-1)
